# Interfacial Charge Transfer Mechanism and Output Characteristics of Identical-Material Triboelectric Nanogenerators

**DOI:** 10.3390/nano15100708

**Published:** 2025-05-08

**Authors:** Lin-Xin Wu, Shi-Jia Ma, Meng-Jie Li, Xian-Lei Zhang, Gang Zheng, Zheng Liang, Ru Li, Hao Dong, Jun Zhang, Yun-Ze Long

**Affiliations:** 1 Shandong Key Laboratory of Medical and Health Textile Materials, Collaborative Innovation Center for Nanomaterials & Devices, College of Physics, Qingdao University, Qingdao 266071, China; wulinxin@qdu.edu.cn (L.-X.W.); mashijia@qdu.edu.cn (S.-J.M.); limengjie1@qdu.edu.cn (M.-J.L.); zhangxianlei@qdu.edu.cn (X.-L.Z.); zhenggang@qdu.edu.cn (G.Z.); liangzheng@qdu.edu.cn (Z.L.); liruqddx@qdu.edu.cn (R.L.); donghao1@qdu.edu.cn (H.D.); 2 Instrumental Analysis Center, Qingdao University, Qingdao 266071, China

**Keywords:** triboelectric nanogenerator, identical materials, piezoelectric testing

## Abstract

When testing the output of piezoelectric devices under different pressures, the friction between the pressure platform and the device causes a large amount of frictional electrical signals to be mixed in the output piezoelectric signal, seriously affecting the measurement accuracy of the piezoelectric signal. The current solution is to encapsulate the contact interface with identical materials to suppress triboelectric interference. However, this work has shown that even when contact separation is implemented at the interface of same media, triboelectric signals can still be generated. The heterogeneous potential distribution of the same material in contact separation has been discovered for the first time through the contact interface potential distribution, proving that charge transfer still exists between the same materials. Atomic force microscopy (AFM) was used to analyze the microstructure of the interface, and it was found that the existence of the surface tip structure would enhance the electron loss. Based on this, a new electron transfer model for surface–tip electron cloud interaction is proposed in this work. In addition, by comparing the output voltage characteristics of the triboelectric nanogenerators (TENGs) of seven polymer materials (e.g., polypropylene (PP), polyethylene (PE), polyvinyl chloride (PVC), polytetrafluoroethylene (PTFE), polyoxymethylene (POM), polyimide (PI), and polyethylene terephthalate (PET)), it was found that the open circuit voltage of PP material was only 0.06 V when they friction with each other, which is 2–3 orders of magnitude lower than other materials. When PP materials are applied to the package of piezoelectric devices, the precision of piezoelectric output characterization can be improved significantly, and a new experimental basis for a triboelectric theory system can be provided.

## 1. Introduction

As a breakthrough technology in the field of mechanical energy harvesting and self-powered sensing, triboelectric nanogenerators (TENGs) have attracted a lot of attention due to their energy conversion efficiency of up to 85%. Their working principle is derived from the contact electrogenesis effect: when two materials with different electron affinity contact, the spontaneous transfer of electrons at the interface is triggered by the difference in work function, and the continuous transformation of mechanical energy into electrical energy is achieved through periodic contact–separation motion. It is widely used in the fields of Wiener energy harvesting, self-powered sensing, and blue energy, in which Song et al. successfully achieved battery power by using polyurethane (PU) and perfluoroalkoxy (PFA) friction, avoiding the risk of replacing batteries with implanted devices [1]. Some researchers realized environmental energy collection by optimizing material friction sequence, and realized intelligent monitoring by using the strong correlation between output electrical signals and environmental parameters [2,3,4,5,6,7,8,9]. Yu et al. [10,11,12,13,14,15] significantly enhanced the output performance of TENGs by optimizing triboelectric layer materials, and successfully applied them to various fields, including sensors, thereby advancing the development and applications of TENG technology. In blue energy harvesting, some researchers [16,17,18,19,20,21] developed an ocean wave energy harvesting system based on the solid–liquid interface friction effect. By optimizing the structure, some researchers [22,23,24,25,26,27,28] significantly improved the low-frequency energy collection efficiency. 

In the existing studies, the friction layer of the TENG is mostly composed of heterogeneous materials, so when the friction medium is changed to the identical material, can the TENG produce output? Few people have explored it. Wang et al. [29] explored the difference in the ability to gain and lose electrons due to curvature differences in the surface during friction of the identical curved material, and proposed a charge transfer model related to curvature. It lays a theoretical foundation for the friction study of planar identical flat materials.

In this work, the triboelectric effect existing in the friction process of the identical planar thin films (self-friction) was investigated through the contact separation mode, and it was confirmed for the first time that a TENG with the same friction layer material (identical materials-based TENG, I-TENG) can generate circuit current, where the peak current output of PI material can reach 106 nA during the friction process, which improves the traditional triboelectric theory. In a further study, we confirmed the universality of this phenomenon by the self-friction of a variety of identical flat film materials. Based on this, this study reveals that the triboelectric interference of piezoelectric output still exists even when the contact surface and the piezoelectric device are packaged with the identical material during the electrical signal output test. In the self-friction test of common polymer materials, it was found that the self-friction output of PP material is only 0.06 V, which can be effectively used as a packaging material to reduce the impact of triboelectricity on piezoelectric signals.

## 2. Materials and Methods

### 2.1. Materials of I-TENG

The friction materials used above are purchased from Dongguan Lingmei New Materials Co., Ltd. (Dongguan, China). These include polypropylene (PP), polyethylene (PE), polyvinyl chloride (PVC), polytetrafluoroethylene (PTFE), polyoxymethylene (POM), polyimide (PI), and polyethylene terephthalate (PET), with specifications of 5 cm × 5 cm and thickness of 0.4 mm. These were attached to the back of an electrode 0.04 mm with commercial conductive copper tape. The materials used in the process of exploring self-friction were all attached in adjacent areas of the identical material, which greatly ensured the consistency of materials.

### 2.2. The Methods of I-TENG Electrical Property Measurement and Material Surface Potential Measurement

LP-3 linear motor was used to realize the contact separation process for I-TENG, and different electrical outputs of I-TENG were explored by applying different pressures. The measuring equipment used was an oscilloscope (KEYSIGHT InfiniiVision DSOX3024T, Silicon Valley, CA, USA) and a type 6514 electrometer (Keithley, Cleveland, OH, USA). In the characterization of the surface potential, a hand-held electrometer was used to measure the surface potential at 25 mm of the normal direction of the sample. An atomic force microscope (AFM, PF-KPFM, Bruker, Germany) was used to characterize the surface potential of materials.

## 3. Results and Discussion

### 3.1. Self-Friction Effect of Identical Materials and Basic Principle of I-TENG

**Figure 1:**
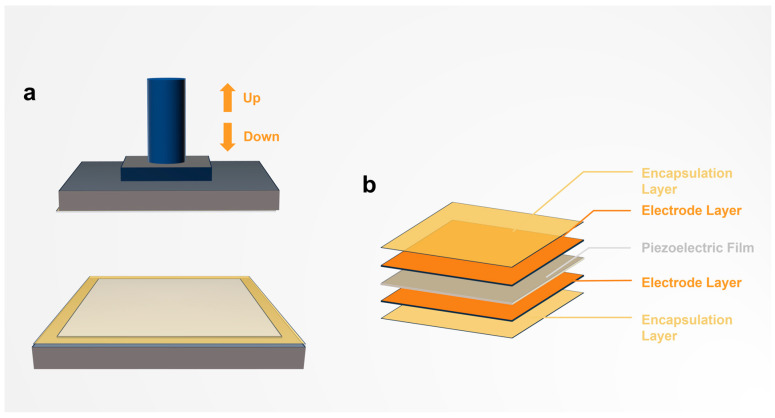
The device and the package structure of the material during the electrical output test of the piezoelectric material. (**a**) The pressure loading platform was explored in terms of how different pressures along the arrow affect the output of piezoelectric materials. (**b**) Packaging structures are commonly used when the output performance of pressurized electrical materials is tested.

As shown in Figure 1a, researchers often use pressure platforms to test the piezoelectric performance of piezoelectric materials under different conditions, but in the process cannot avoid the mutual friction of the contact surface. In order to reduce the influence of triboelectricity on the piezoelectric output performance, most researchers will use the identical material to encapsulate the contact surface and the piezoelectric material, which theoretically can avoid the generation of triboelectric signals (Figure 1b). But as we proved in our experiments, even the use of the identical material in packaging does not avoid the generation of frictional electrical signals.

**Figure 2:**
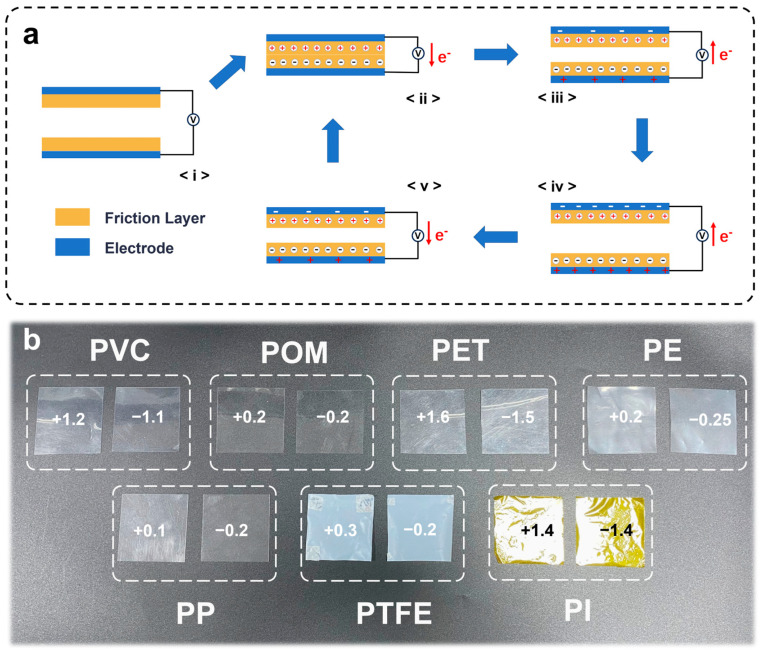
The structure of I-TENG and the surface potential of the planar film materials after friction. (**a**) The specific structure and schematic diagram of I-TENG. (**b**) The surface potential changes of seven common polymer materials after self-friction.

To explore this process, we fabricated a contact separation mode I-TENG using seven commonly used polymer materials. Compared with conventional TENG, the two friction layers of I-TENG are identical materials. As shown in Figure 2a, electrodes are attached on both sides of the friction layer to sense the change in electric potential between the two friction layers and form a loop. Before the contact of the friction layer, its surface potential is zero (Figure 2(ai)). When there is an external displacement, the two friction layers contact each other and friction occurs, and electron transfer occurs (Figure 2(aii)). At this time, because the charge generated by friction is limited to the surface of the friction layer, when two equal amounts of opposite charges coexist on the identical plane, there is no induced charge on the electrode. In the process of the gradual separation of the two friction layers, equal amounts of opposite charges are generated in the two electrodes due to electrostatic induction, resulting in a potential difference in the closed loop (Figure 2(aiii)). When the two friction layers are gradually separated under the action of external forces, the open circuit voltage at both ends of the electrode will also increase. When the resistance value of the voltmeter is close to the ideal value, the voltage will continue to increase until it is stable. Before the friction layers are recombined, the voltage at both ends will remain at the peak (Figure 2(aiv)). Then, as the friction layers gradually approach, the electric potential on both sides of the electrode gradually decreases, and when the friction layer returns to the state shown in Figure 2(aii), the surface electric potential on both sides of the electrode disappears.

In order to further verify that the output of the device is produced by friction of the identical material, we take two 5 × 5 cm^2^ square samples in adjacent areas on seven identical materials and flush the surface charge with deionized water. After heating and drying, a process to eliminate the static charges of the sample, the use of an electrostatic tester at a distance of 25 mm from the sample measured a surface potential of zero. After self-friction of the seven materials, the surface potential is shown in Figure 2b. Through experiments, we found that identical materials all have charge transfer in the process of contact separation friction.

Polyethylene terephthalate (PET) is the most obvious material in this process. The two surfaces of the identical materials with opposite electrical properties were measured by an electrostatic tester at a distance of 25 mm apart, and their potential was +1.6 kV and −1.5 kV, respectively, and the potential difference between the two surfaces reached 3.1 kV. In contrast, PP material in this process yielded a surface potentials of the positive and negative sides of +0.1 kV and −0.2 kV, and the potential difference was only 0.3 kV. In addition, we left the rubbed material for a period of time until the surface potential was zero, continued to wash the surface with deionized water and heat it, and after rubbing again, the surface potential was identical to the result of the first test.

### 3.2. Study on Effect Mechanism of Self-Friction of Identical Materials

Through the above experiments, we confirm that the identical materials do exhibit electron transfer in the process of self-friction, resulting in the opposite surface potential of the two friction layers. Taking the PET film as an example, we further characterized the surface atomic morphology of the planar friction layer by using atomic force microscopy (AFM).

**Figure 3:**
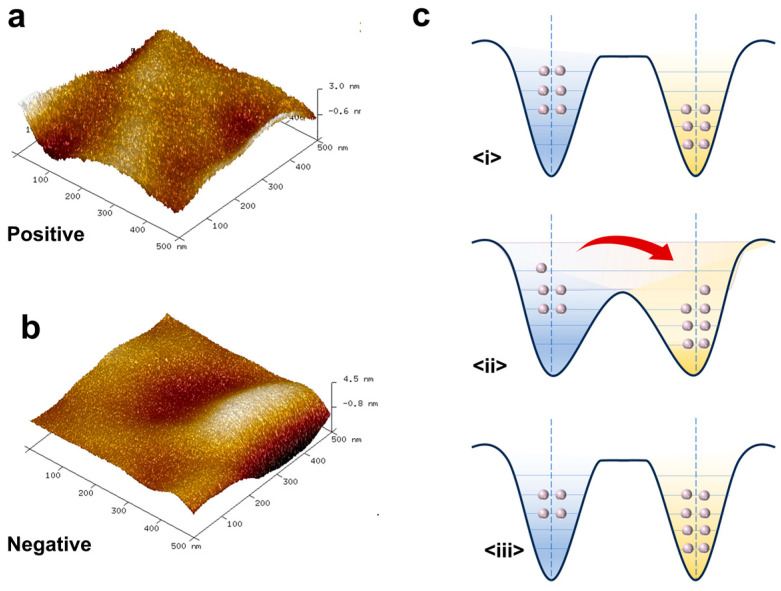
Mechanism of self-friction effect. (**a**) AFM topography of PET flat film with positive surface potential. (**b**) AFM topography of PET flat film with negative surface potential. (**c**) Electron cloud well model.

As shown in Figure 3a,b, in the microstructure, the surface with positive charge has more pointed protrusions than the surface with negative charge. In addition, after grinding the tip flat by mechanical grinding, we found that the ability to lose electrons was significantly reduced. Based on the above experimental results, we have proposed a cloud well model on the basis of the curved surface self-friction model proposed by Wang [29] to explain the mechanism of contact electricity generated by the same flat material. In conventional wisdom, triboelectricity is always generated by contact between two different materials. However, in this experiment, triboelectricity was also generated after contact separation between identical planar materials with identical chemical properties, which may be determined by the microstructure of the surface of the planar materials. As shown in Figure 3(ci), the atomic arrangement of the tip may be distorted due to curvature, resulting in changes in the surface electron state density, so that the electrons of the tip atom have higher surface energy. In Figure 3(ci), the two atoms remain independent of each other until they are in contact, and the electron clouds do not overlap. At the same time, the existence of the potential well prevents the overflow of electrons, making the atom relatively stable. When two surfaces come close to each other and touch due to mechanical forces, the electron clouds overlap and create ionic or covalent bonds. Since the electrons at the tip of the atom have a higher potential energy, the electrons are easily transferred from the higher to the lower energy orbital (Figure 3(cii)). Through this process, electrons are transferred from the tip atoms to the smooth atoms, resulting in a difference in the amount of charge on the two surfaces. According to this mechanism, the phenomenon that the tip electron is easily lost in the microstructure can be well explained. In addition, the output difference of I-TENG in the self-friction process of different materials may be determined by the work function of the material itself, which still needs further research.

### 3.3. Output Properties of I-TENG for Different Polymer Materials

**Figure 4:**
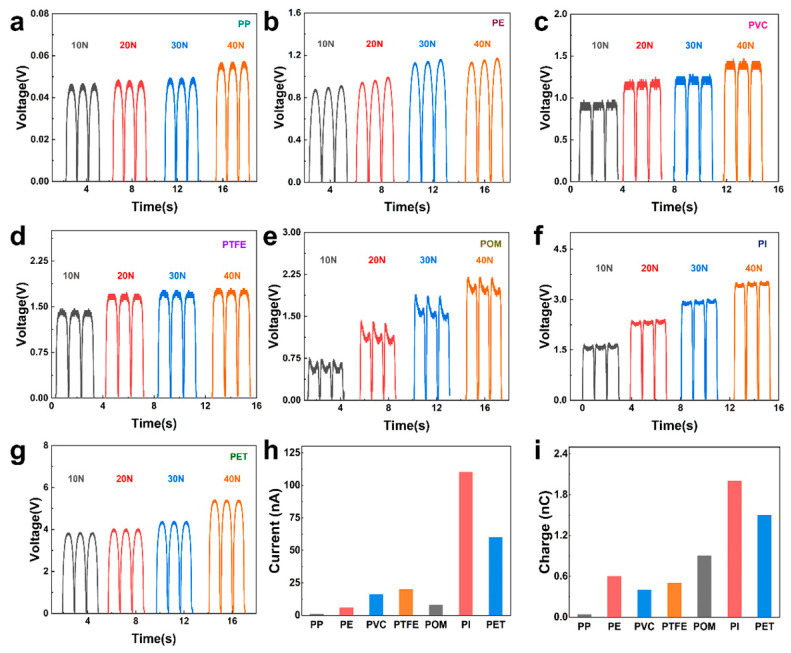
I-TENG output of different materials. (**a**–**g**) Voltage output of I-TENG after self-friction of PP, PE, PVC, PTFE, POM, PI, and PET flat films, respectively. (**h**) Short-circuit currents of I-TENG of different materials. (**i**) Short-circuit charge transfer of I-TENG of different materials.

Based on the above conclusions, in order to reduce the influence of triboelectricity in piezoelectric materials, as shown in Figure 4, the output of I-TENG with seven different materials under different pressures was tested. Among them, the PP film has the lowest open-circuit voltage in the process of self-friction, only 0.06 V (Figure 4a). In Figure 4a–g, the voltage output increases successively. For the flat PET material, the output of I-TENG is the largest, and the output open-circuit voltage reaches 6 V under the pressure of 40 N. In addition, the measured output current (Figure 4h) and transferred charge (Figure 4i) also satisfy the same law.

### 3.4. The Application of Polypropylene (PP) Materials in Piezoelectric Testing

To effectively eliminate interference from the triboelectric effect in piezoelectric testing, identifying a packaging material capable of suppressing triboelectric charge transfer has become a key research focus.

The experimental data demonstrate that, compared to other common packaging materials, polypropylene (PP) exhibits significant advantages during self-friction processes: its triboelectric layer potential difference, I-TENG output voltage, current, and transferred charge quantity all reach the lowest values—far below the output levels of conventional piezoelectric materials—making it an ideal packaging medium for piezoelectric applications.

**Figure 5:**
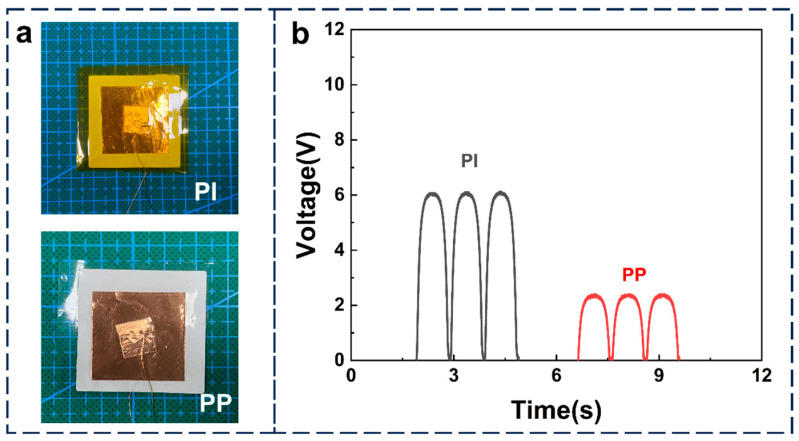
The application of polypropylene (PP) materials in piezoelectric testing. (**a**) Images of the same piezoelectric film encapsulated with PI and PP materials, respectively. (**b**) The output voltage of the piezoelectric film encapsulated with PI and PP materials under periodic pressure (20 N, 1 Hz).

For a direct comparison of performance differences among packaging materials, Figure 5a presents a contrast test between polyimide (PI) and PP encapsulating the same piezoelectric material. Under a periodic 20 N compressive force, the PI-packaged piezoelectric sample showed a substantial triboelectric contribution in its output signal, whereas PP encapsulation effectively reduced such interference (Figure 5b).

### 3.5. The Application of I-TENG in Self-Powered Stride Sensors

**Figure 6:**
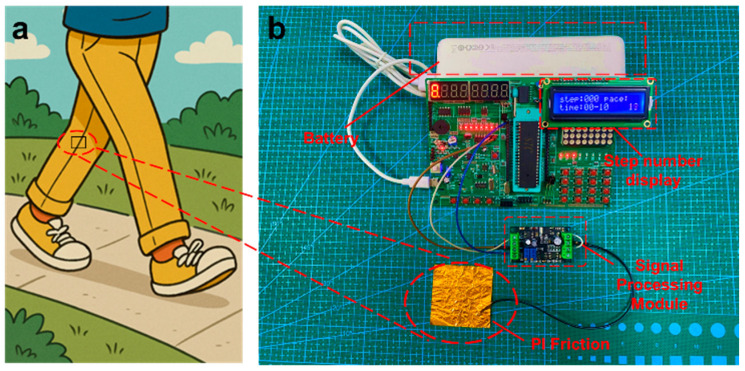
The application of I-TENG in self-powered stride sensors. (**a**) Concept map of the application of I-TENG in self-powered stride sensors. (**b**) The physical image of I-TENG used for detecting stride movement.

Triboelectric Nanogenerators (TENGs) can be applied in human motion monitoring. Traditional monitoring systems must continuously supply power to sensors in order to capture people’s movement states in real time, which consumes a large amount of electrical energy and shortens the battery life of the monitoring system. Although the output voltage in Figure 4 is very small, we can apply it to the self-powered stride monitoring sensor. By converting the mechanical energy generated by movement into electrical energy, it can be used to analyze the movement state of the human body. As shown in Figure 6a, when people are walking, the trouser legs rub against each other. The frictional charges generated can be converted into electrical signals through the I-TENG, and the movement state of the wearer can be analyzed by the single-chip microcomputer. As shown in the demonstration experiment in Figure 6b, when the PI materials rub against each other, the output electrical signals can be collected by the single-chip microcomputer and used to analyze and obtain the stride length of the movement (Movie S1 in the Supplementary).

## 4. Conclusions

In summary, we further explore the triboelectricity generation process of the identical flat materials. By rubbing the identical flat materials, I-TENG produces voltage output while there are opposite charges between the two contact surfaces, which further proves that the process is caused by charge transfer. Furthermore, in the microstructure characterization of the contact surface, we found that a contact surface with a large number of tips is more likely to lose electrons, and the ability to lose electrons is rapidly weakened after the tips are smoothed. Based on the above conclusions, we introduce the concept of surface states into the experiment, and propose a model of electron transition based on surface tip atoms to further illustrate the whole process. Subsequently, we compared the self-friction output voltage of seven common packaging materials, and found that the self-friction output open circuit voltage of PP material is only 0.06 V, which can be effectively used in piezoelectric material packaging.

## Data Availability

Data are contained within the article and Supplementary Materials.

## Supplementary Materials

The following supporting information can be downloaded at: https://www.mdpi.com/article/10.3390/nano15100708/s1, Movie S1: The application of I-TENG in self-powered stride sensors.
